# *In vivo* genetic manipulation of inner ear connexin expression by bovine adeno-associated viral vectors

**DOI:** 10.1038/s41598-017-06759-y

**Published:** 2017-08-04

**Authors:** Giulia Crispino, Fabian Galindo Ramirez, Matteo Campioni, Veronica Zorzi, Mark Praetorius, Giovanni Di Pasquale, John A. Chiorini, Fabio Mammano

**Affiliations:** 1 Venetian Institute of Molecular Medicine, Foundation for Advanced Biomedical Research, Padua, Italy; 2 Department of Physics and Astronomy “G. Galilei”, University of Padua, Padua, Italy; 3 Department of Biomedical Sciences, Institute of Cell Biology and Neurobiology, Italian National Research Council, Monterotondo, RM Italy; 4 Department of Otolaryngology, University of Heidelberg Medical Center, Heidelberg, Germany; 5 Molecular Physiology and Therapeutics Branch, National Institute of Dental and Craniofacial Research, National Institutes of Health, Bethesda, MD USA; 6 Shanghai Institute for Advanced Immunochemical Studies, ShanghaiTech University, Shanghai, 201210 China; 7 Present Address: Physiology Institute, Autonomous University of Puebla, Puebla, Mexico; 8 Present Address: Department of Biochemistry and Biotechnology, University of Pavia, Pavia, Italy

## Abstract

We have previously shown that *in vitro* transduction with bovine adeno–associated viral (BAAV) vectors restores connexin expression and rescues gap junction coupling in cochlear organotypic cultures from connexin–deficient mice that are models DFNB1 nonsyndromic hearing loss and deafness. The aims of this study were to manipulate inner ear connexin expression *in vivo* using BAAV vectors, and to identify the optimal route of vector delivery. Injection of a BAAV vector encoding a bacterial Cre recombinase via canalostomy in adult mice with floxed connexin 26 (Cx26) alleles promoted Cre/LoxP recombination, resulting in decreased Cx26 expression, decreased endocochlear potential, increased hearing thresholds, and extensive loss of outer hair cells. Injection of a BAAV vector encoding GFP-tagged Cx30 via canalostomy in P4 mice lacking connexin 30 (Cx30) promoted formation of Cx30 gap junctions at points of contacts between adjacent non-sensory cells of the cochlear sensory epithelium. Levels of exogenous Cx30 decayed over time, but were still detectable four weeks after canalostomy. Our results suggest that persistence of BAAV-mediated gene replacement in the cochlea is limited by the extensive remodeling of the organ of Corti throughout postnatal development and associated loss of non-sensory cells.

## Introduction

Up to 50% of prelingual hearing impairment is linked to the DFNB1 locus on chromosome 13q11–q12^1^, which comprises the genes encoding two structurally and functionally related gap junction proteins, Cx26 (*GJB2*) and Cx30 (*GJB6*)^2, 3^. In mice, the coordinated expression of Cx26 and Cx30^4, 5^ has been shown to depend on the spacing of their surrounding chromosomal region^6^. Cx26 and Cx30 colocalize in supporting and epithelial cells of the organ of Corti, in basal and intermediate cells of the stria vascularis, and in type 1 fibrocytes of the spiral ligament^7–9^, forming vast gap junction networks that can be visualized directly, using voltage imaging, even during the first postnatal week^10^. Gap junctions are dynamic structures and connexins turn over rapidly, with half lives of 2–5 hours^11, 12^.

Epithelial and supporting cells of the cochlear sensory epithelium (hereafter collectively referred to as non-sensory cells) provide trophic and mechanical support to sensory hair cells, which perform mechanotransduction^13^, i.e. the conversion of sound-evoked mechanical stimuli applied to their hair bundle into graded changes of their membrane potentials known as receptor potentials^14^. Mechanotransduction and generation of receptor potentials depend on the potential difference, known as endocochlear potential, between endolymph (a K^+^ rich fluid that fills scala media) and perilymph (a Na^+^ rich fluid akin to cerebrospinal fluid that fills scala tympany and scala vestibuli)^15, 16^. The indispensable endocochlear potential, which in mice exceeds 100 mV^17^, is generated by the activity of gap-junction coupled cells in the lateral wall (spiral ligament and stria vascularis)^18, 19^ and provides the driving force required for K^+^ influx from endolymph to hair cell cytosol via mechanically activated channels in the hair bundle^20, 21^. Another essential prerequisite for generating receptor potentials is electrical insulation of the hair cells from the rest of sensory epithelium^22^. Consistent with this requirement, neither the outer nor the inner hair cells express any connexin^23–25^. Inner hair cell depolarization drives glutamate release at their synaptic pole and consequent excitation of afferent neurotransmission along the auditory nerve^26–28^. Outer hair cell receptor potentials activate prestin in their basolateral membrane^29–32^ supporting mechanical amplification of basilar membrane movements^33, 34^ and increasing hearing sensitivity and frequency selectivity^35, 36^.

Virus−mediated gene delivery is largely used for human gene therapy, and clinical trials are being carried out to treat cancer, cardiovascular disorders, monogenic disorders, neurological disorders^37^, and eye diseases^38^. Gene delivery to the mammalian inner ear has been performed with a variety of viral vectors, including herpes simplex type I virus and vaccinia virus, lentiviruses, retroviruses and adenoviruses, with limited success and several shortcomings^39–41^. Furthermore, an important component of a future therapeutic intervention plan is the optimal route of vector delivery to the inner, and this has not been uniquely identified yet^42–47^.

Gene delivery studies with adeno–associated virus (AAV) were performed in mice *in utero* ^48, 49^, as well as in young guinea pigs^50–52^. Subsequent work provided proof-of-principle evidence that intracochlear delivery of AAV vectors can mediate restoration of hearing in mice lacking vesicular glutamate transporter 3 in inner hair cells (VGLUT3)^53^ and partial restoration in mice lacking transmembrane channel–like 1 (TMC1), a protein known to affect the permeation properties of sensory transduction channels in both inner and outer hair cells^54^. However, outer hair cell function was not restored in that study. Recently, a synthetic adeno-associated viral vector (Anc80L65) with high tropism for both types of hair cells was delivered via round window membrane injections to treat a knock-in mouse model for Usher syndrome type 1 C^55^.

Here, we used BAAV vectors^4, 5, 49, 56–58^ to deliver reporter and connexin genes to the inner ear of adult as well as neonatal mice. Our results in wild type and transgenic mice indicate that BAAV, administered via canalostomy^42, 46, 59–61^, is highly effective in transducing cochlear non−sensory cells *in vivo*, however the expression level of the transgene is insufficient to sustain adequate connexin expression from postnatal development to adulthood.

## Results

### Canalostomy is a viable and safe route for gene delivery to the mouse cochlea

**Figure 1:**
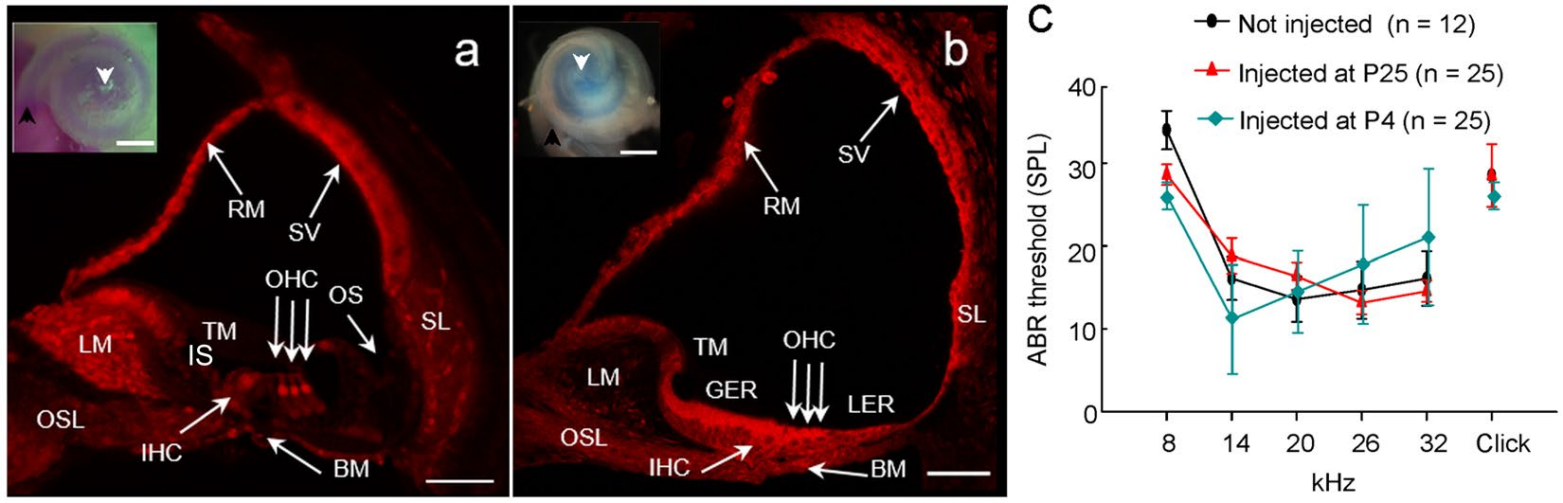
*In vivo* fluid delivery via canalostomy to mouse cochlea. (**a,b**) Midmodiolar cochlear sections from P25 (**a**) and P4 (**b**) mice injected with fluorescent wheat germ agglutinin (which labels cell membranes) examined by confocal microscopy; scale bars: 50 μm. Insets: representative light microscopy images of cochleae from P25 (**a**) or P4 (**b**) mice injected with bromophenol blue, dissolved in PBS; images were acquired 5 min after canalostomy; scale bars: 500 μm; arrowheads point to the base (black) and apex (white) of the cochlea. BM: basilar membrane; GER, greater epithelial ridge; IHC: inner hair cell; IS: inner sulcus; LER, lesser epithelial ridge; LM: spiral limbus; OHC: outer hair cells; OS: outer sulcus; OSL, osseous spiral lamina; RM: Reissner’s membrane; SL: spiral ligament; SV: stria vascularis; TM: tectorial membrane. (**c**) Hearing thresholds determined by analysis of auditory brainstem responses (ABR) in wild type mice injected at P25 (red), P4 (cyan) and non-injected controls (black); ordinates are sound pressure levels (SPL) relative to 20 µPa: Error bars represent standard error of the mean (s.e.m.).

To validate canalostomy as a potential route to target cochlear structures, we delivered bromophenol blue or fluorescent wheat germ agglutinin dissolved in PBS in the semicircular canal of P25 or P4 wild type mice (C57BL6/N), resulting in widespread distribution of the injected agent without visible damage to cochlear duct structures (Fig. 1a,b). In a subsequent set of experiments, we injected Dulbecco’s Modified Eagle Medium/Nutrient Mixture F−12 (DMEM/F12) via canalostomy in wild type mice at P25 (2.5 μl) or P4 (1.0 μl). Four weeks later, we assessed hearing performance by recording auditory brainstem responses (ABRs), which are electrical signals evoked from the brainstem following the presentation of sound stimuli^62^. We measured the IV wave thresholds of the ABR for click and tone burst stimuli of 8, 14, 20, 26, 32 kHz and found no differences in injected mice compared with non injected controls (Fig. 1c). These results indicate that delivery of DMEM/F12 to the inner ear via canalostomy causes no adverse effects on hearing and could function as an effective route of vector delivery.

### *In vivo* transduction with BAAV vectors via canalostomy achieves widespread expression of a reporter gene in non-sensory cells of the mouse cochlear duct

**Figure 2:**
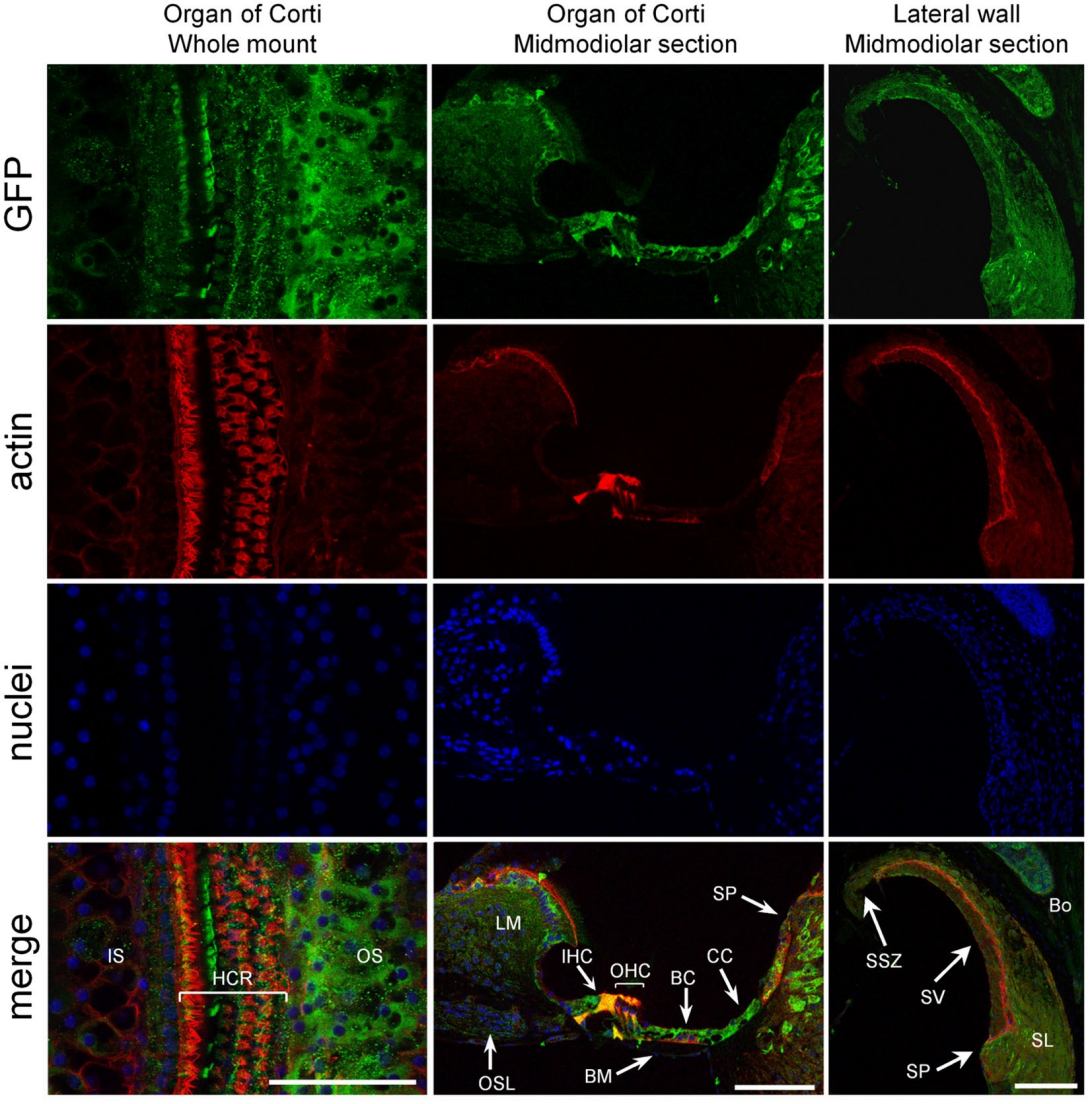
Confocal immunofluorescence imaging of cochlear cross–sections from mice injected at P25 with BAAVβ-actin-GFP. Color code: β-actin-GFP, green; actin filaments, red; nuclei, blue. BC: Böttcher cells; BM: basilar membrane; Bo: bone; HCR: hair cell region; IHC: inner hair cell; IS: inner sulcus; LM: spiral limbus; OHC: outer hair cells; OS: outer sulcus; OSL: osseus spiral lamina; SL: spiral ligament; SP: spiral prominence; SSZ: supra-strial zone; SV: stria vascularis. Scale bars: 50 μm.

To evaluate the efficacy of *in vivo* transduction via canalostomy, we delivered a previously tested reporter gene vector, BAAVβ−actin−GFP^56, 58^ prepared in DMEM/F12, to the inner ear of P25 wild type mice. Four weeks later, we processed the cochlea for confocal immunofluorescence imaging (Fig. 2). No β−actin−GFP signal was detected in cochlear sensory hair cells, whereas diffuse expression was clearly visible in spiral limbus and spiral prominence, two structures populated by fibrocytes, as well as in non−sensory cells of the cochlear sensory epithelium. In particular, the majority of Hensen’s and Claudius cells, some pillar cells and inner sulcus cells expressed β−actin−GFP. Extensive transgene expression was also evident in the lateral wall of the cochlea, namely in the spiral ligament, stria vascularis and supra-strial zone (Fig. 2).

Prior work in adult guinea pigs used cochleostomy as route of administration and reported a higher efficiency of expression of BAAVβ-actin-GFP when delivered into scala media compared to a scala tympani approach; however the scala media approach resulted in hair cells loss^58^. We replicated these experiments in mice injected at P25 and noted expression of the transgene not only in scala media but also in scala tympani and scala vestibuli, accompanied by an alteration of the cochlear structure and the Reissner’s membrane, highlighting the limitation of this technique for clinical applications (Figure S1).

Altogether, these experiments suggest canalostomy as a preferred route for BAAV-mediated transgene delivery to the mouse cochlea.

### BAAV–driven Cre–Lox recombination abates Cx26 in the cochlea of adult Cx26^loxP/loxP^ mice

**Figure 3:**
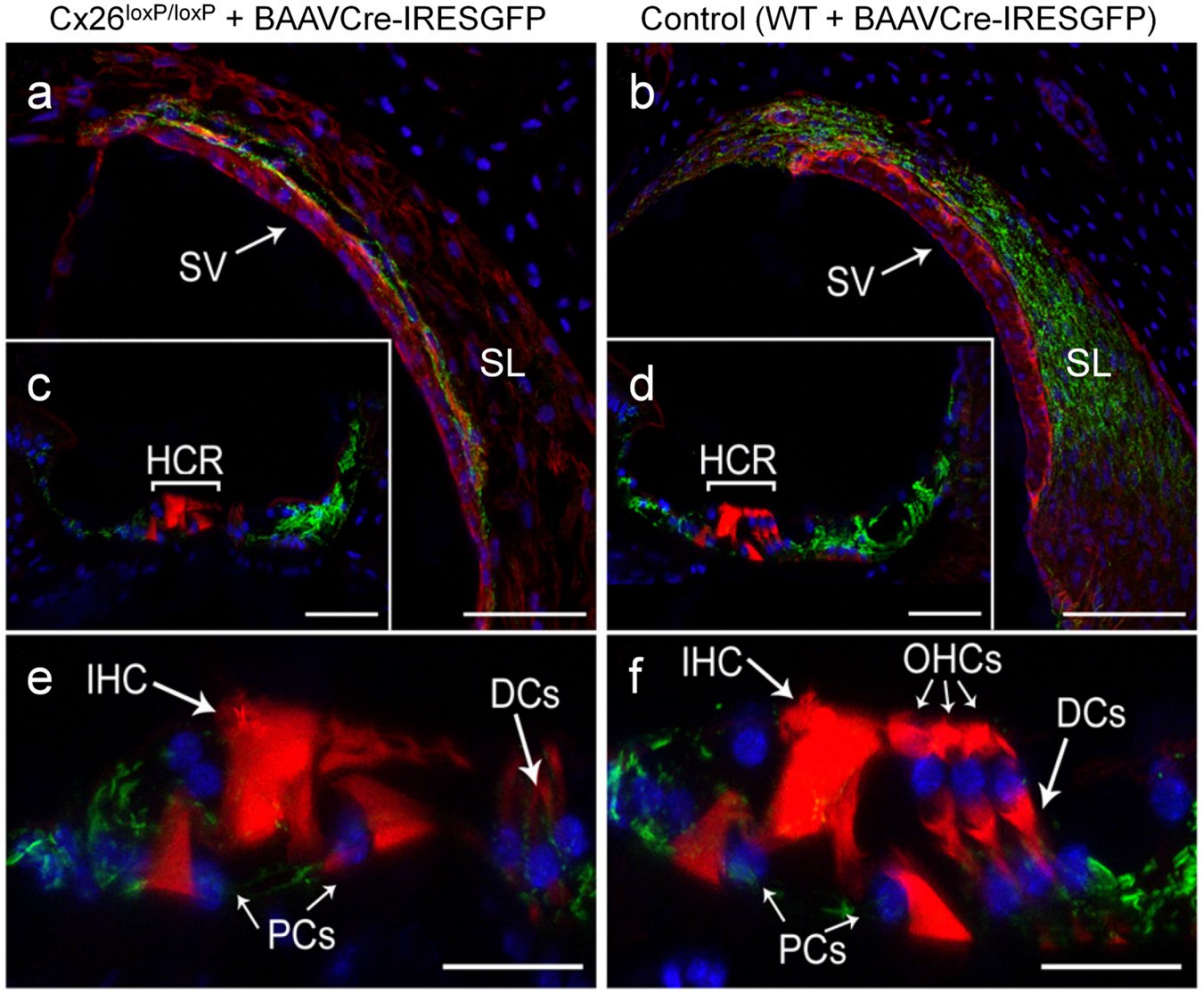
Confocal immunofluorescence imaging of cochlear cross–sections from mice injected at P25 with BAAVCre–IRESGFP viral vectors. Color code: Cx26, green; actin filaments, red; nuclei, blue. (**a,b**) Stria vascularis (SV) and spiral ligament (SL). (**c,d**) organ of Corti; HCR, hair cell region. (**e,f**) close–up view of the HCR from c,d, respectively. DCs: Deiters’ cells; IHC: inner hair cell; OHCs: outer hair cells; PCs: pillar cells. Scale bars: 50 μm in (**a–d**); 25 μm in (**e,f**).

**Figure 4:**
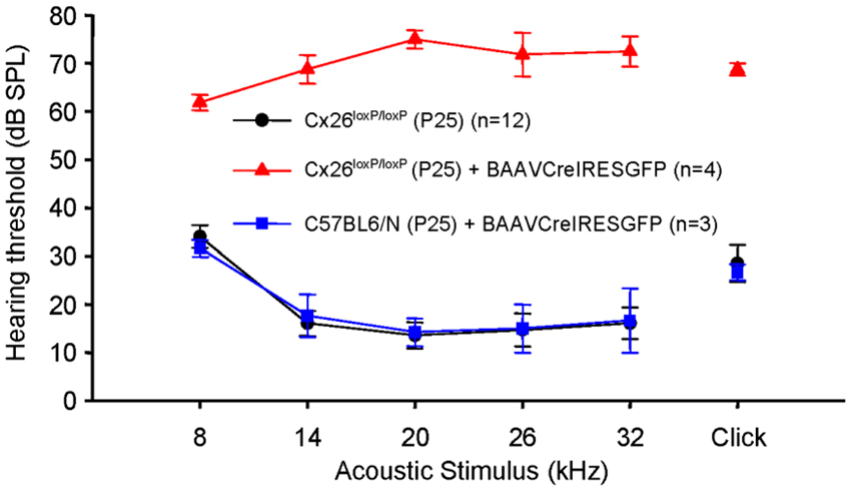
Hearing thresholds determined by analysis of auditory brainstem responses (ABR) in mice injected with BAAVCre–IRESGFP and their respective controls. Error bars represent s.e.m.

Inner ear connexins play a crucial developmental role and are essential for the maturation of sensory hair cells, despite the fact that hair cells do not express any connexin^63^. The conventional gene knockout approach is unsuitable for postnatal studies on Cx26 because homozygous knockout mice die in utero due to insufficient transplacental uptake of glucose^64^. Here, we investigated the role of Cx26 in the maintenance of sensory cells utilizing BAAV for the timed and localized knockout of Cx26 based on the Cre/loxP system^65, 66^ using a canalostomy route of delivery. Cre-Lox recombination is an irreversible process which does not require sustained protein expression, as it needs to take place only once and requires a limited amount of Cre recombinase^65, 66^. We engineered a BAAV vector encoding GFP-tagged bacterial Cre recombinase under the CMV promoter (BAAVCre-IRESGFP). We prepared the vector in DMEM/F12 and injected it via canalostomy to the inner ear of P25 Cx26^loxP/loxP^ mice. Confocal immunofluorescence imaging of cochlear midmodiolar sections obtained four weeks after canalostomy showed BAAVCre–IRESGFP caused a dramatic reduction of Cx26 immunofluorescence signals in the lateral wall of Cx26^loxP/loxP^ mice (n = 4) (Fig. 3a), but had no effect on tissue morphology, cell viability and connexin expression in the inner ear of wild type C57BL/6 N mice (n = 3) used as controls (Fig. 3b). Cre-mediated excision of Cx26 was comparatively less effective in the organ of Corti, as indicated by the presence of residual Cx26 expression (Fig. 3c and d for control). q-PCR analysis of whole cochlea samples confirmed a 43.5 ± 2.2% overall reduction of Cx26 transcript levels with respect to the contra lateral non–injected ear (n = 4; p < 0.001, paired *t* test). Based on these immunofluorescence results, we conclude that the majority of Cx26 loss occurred in fibrocytes of the spiral ligament. Consistent with this conclusion, the endocochlear potential was significantly lower in Cx26^loxP/loxP^ mice injected with BAAVCre–IRESGFP at P25 (26 ± 10 mV, n = 3) compared to non–injected Cx26^loxP/loxP^ controls (106 ± 3 mV, n = 11; p < 0.001, ANOVA). Inner hair cells, pillar cells and Deiters’ cells were maintained in all transversal sections, spanning the whole length of the cochlear duct. Instead, we observed a 92 ± 16% (n = 3) loss of outer hair cells (Fig. 3e and f for control). In agreement with these results, we found remarkably increased hearing thresholds in Cx26^loxP/loxP^ mice injected with BAAVCre–IRESGFP at P25, whereas thresholds in C57BL/6 N mice injected at P25 with BAAVCre–IRESGFP were indistinguishable from non–injected Cx26^loxP/loxP^ controls (Fig. 4). Therefore, we conclude that: (1) the Cre recombinase encoded by the BAAV vector and delivered via canalostomy acted specifically on the floxed alleles; (2) BAAV-based vectors permit efficient manipulation of floxed genes in the mammalian inner ear *in vivo*; (3) survival of cochlear outer hair cells depends on Cx26 expression in the lateral wall.

### Cx30 expression is restored in the cochlea of Cx30^Δ/Δ^ mice following *in vivo* delivery of BAAVCx30GFP via canalostomy at P4

In the experiments described above we have demonstrated that BAAV vectors can promote reduction of connexin expression at a specific time point in the cochlea of adult Cx26^loxP/loxP^ mice. Although this is useful to explore the function of inner ear connexins in hearing, restoration of connexin expression in DFNB1 mouse models is clearly of primary importance for future therapeutic applications.

Targeted ablation of Cx26 in the mouse inner ear (Cx26^Sox10Cre^, Cx26^OtogCre^) leads to irreversible cell loss in the organ of Corti either before or around the onset of hearing^5, 67^, which in mice occurs at P12^68^. A similar problem affects also Cx30^−/−^ mice, which show hearing loss at all frequencies accompanied by complete absence of endocochlear potential^69, 70^. Therefore it is clear that any intervention must be scheduled before irreversible damage to the organ of Corti, due to lack of connexin expression, takes place. Previously, we succeeded in restoring connexin expression and rescued intercellular coupling *in vitro*, by transducing cochlear organotypic cultures from P5 Cx26^Sox10Cre^ or Cx30^−/−^ mice with BAAV vectors encoding respectively Cx26 or Cx30^4, 5^. Here, we used a BAAVCx30GFP vector^4^, which encodes GFP-tagged mouse Cx30 under the control of the CMV promoter, prepared in DMEM/F12, and injected it to P4 Cx30^–/–^ mice via canalostomy. Auditory thresholds of injected mice, measured four weeks after surgery, remained super imposable to those of untreated Cx30^–/–^ mice (p < 0.001, ANOVA) (Figure S2a).

The hearing loss phenotype exhibited by Cx30^−/−^ mice depends on the cumulative effect of deletion of Cx30 and 3’ insertion of the lacZ and neo genes^6^, which are associated with dramatically reduced Cx26 levels^4^. Previous work showed restoration of hearing and prevention of hair cell death in Cx30^−/−^ mice in which extra copies of the Cx26 gene were transgenically expressed from a modified bacterial artificial chromosome^71^. Therefore we injected Cx30^–/–^ mice with a BAAV vector espressing Cx26CFP via canalostomy at P4, but also this treatment remained ineffective (Figure S2a). q–PCR analyses using primers specific for GFP tag to monitor transgene expression at 2, 3, 6, and 30 days after canalostomy, showed that transcript levels of Cx26CFP were maximal 2 days after gene delivery and thereafter decreased continuously. Thirty days after canalostomy, transgene expression was only 15.5 ± 4.5% (p < 0.005, ANOVA) of its initial peak (Figure S2b).

**Figure 5:**
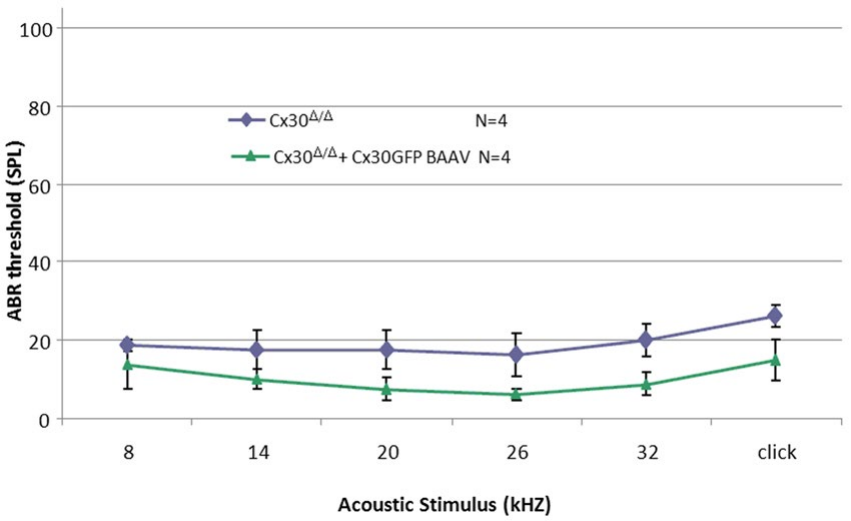
Hearing thresholds of adult Cx30^Δ/Δ^ mice four weeks after delivery of BAAVCx30GFP viral vector at P4 and their respective age-matched non-injected controls. Error bars represent s.e.m.; differences are significant for 32 kHz (p = 0,022) and click responses (p = 0,043) (two tailed t-test).

These negative outcomes might depend, at least in part, on disruption of the cytoarchitecture of the sensory epithelium and the stria vascularis in the Cx30^−/−^ mouse model^69, 70^. Therefore, we decided to also test the Cx30^Δ/Δ^ Cx30 knock out strain. Despite being ubiquitously deprived of Cx30, hearing is normal in Cx30^Δ/Δ^ mice^6^ and their sensory epithelium is well preserved (Figure S3). We administered the BAAVCx30GFP vector prepared in DMEM/F12 to P4 Cx30^Δ/Δ^ pups via canalostomy, and four weeks later we measured ABR thresholds. Compared to non-injected age-matched Cx30^Δ/Δ^ controls, threshold values were comparable or even slightly lower in Cx30^Δ/Δ^ mice injected with BAAVCx30GFP (Fig. 5). Therefore, we conclude that delivery of exogenous Cx30 gene via BAAV and canalostomy does not cause any hearing loss.

**Figure 6:**
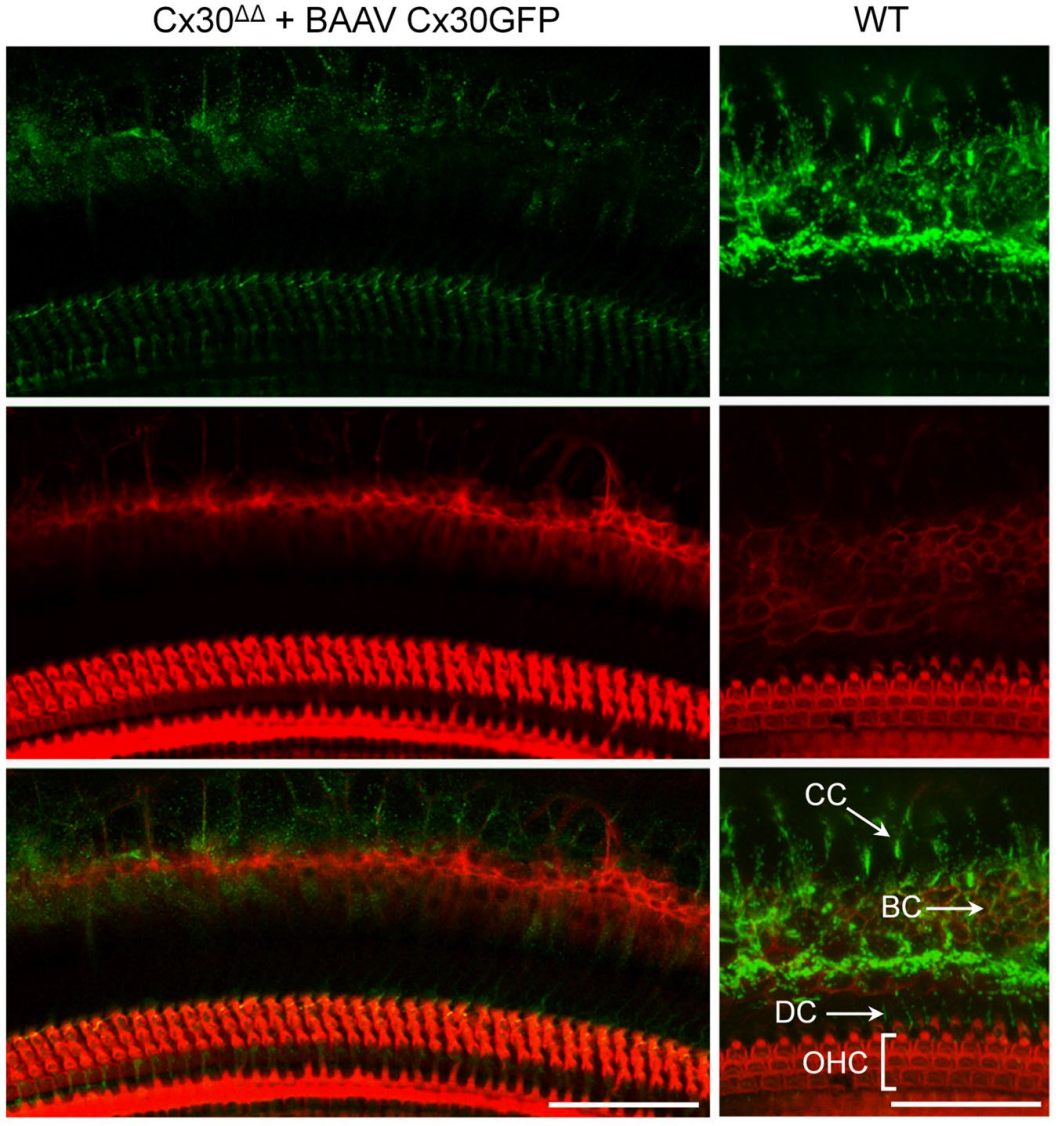
Cx30 expression in the organ of Corti of Cx30^Δ/Δ^ mice four weeks after delivery of BAAVCx30GFP viral vector at P4. Shown are whole mount preparations from injected Cx30^Δ/Δ^ mice (Cx30^Δ/Δ + ^BAAVCx30GFP) and non-injected wild type age-matched controls (WT). The green signal was generated by immunostaining against GFP (Cx30^Δ/Δ^ + BAAVCx30GFP) and native Cx30 (WT), respectively; red, actin filaments; blue, nuclei. BC: Böttcher cells CC: Claudius’ cells; DC: Deiters’ cells; OHC, outer hair cells. Scale bars: 50 μm.

**Figure 7:**
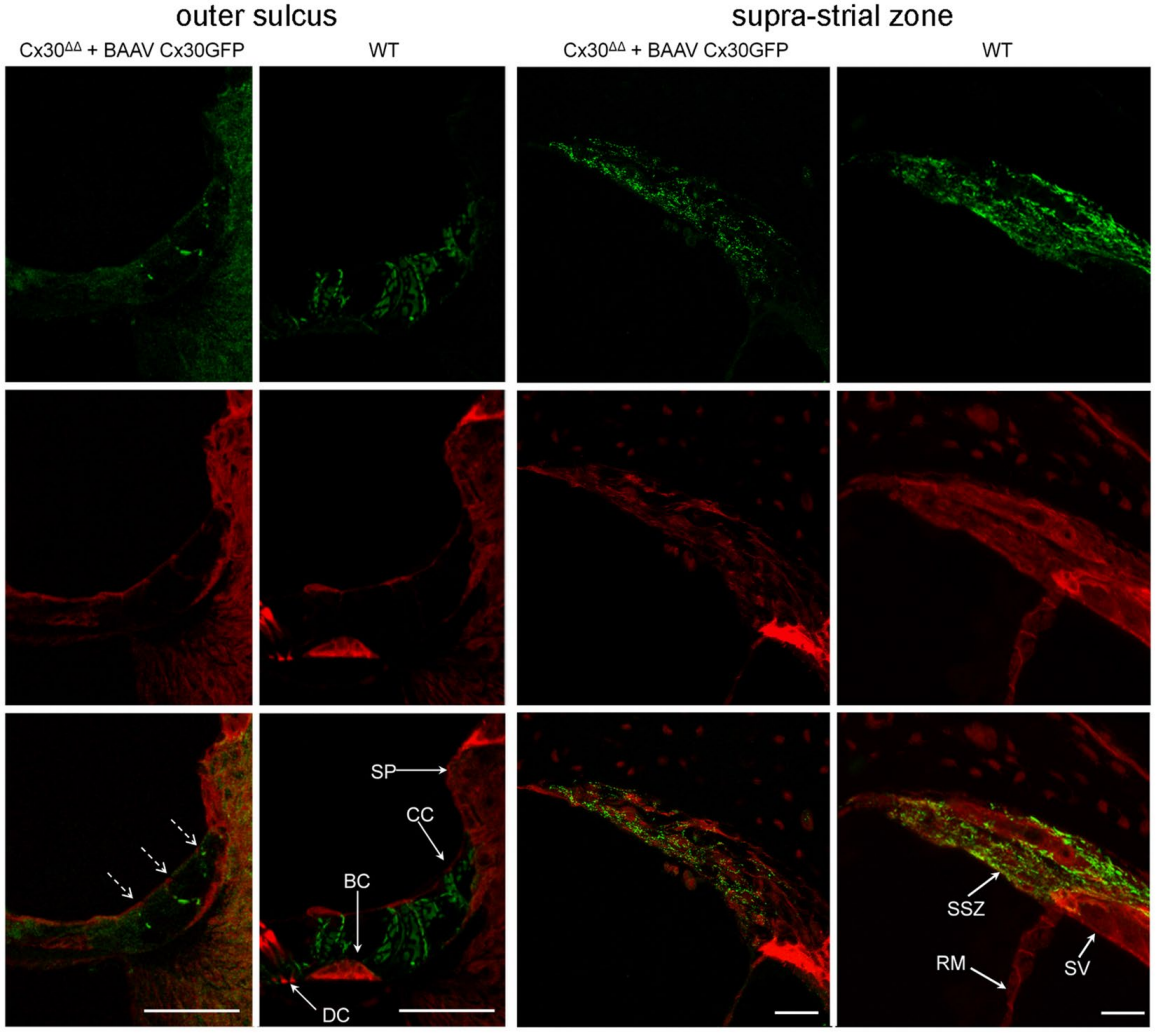
Cx30 expression in cochlear outer sulcus and supra-strial zone of Cx30^Δ/Δ^ mice four weeks after delivery of BAAVCx30GFP viral vector at P4. Shown are midmodiolar sections from injected Cx30^Δ/Δ^ mice (Cx30^Δ/Δ + ^BAAVCx30GFP) and non-injected wild type (WT) age-matched controls. Dashed arrows indicate gap junction plaques formed by recombinant Cx30GFP. The green signal was generated by immunostaining against GFP (Cx30^Δ/Δ+^ BAAVCx30GFP) and native Cx30 (WT), respectively. red, actin filaments; blue, nuclei. BC: Böttcher cells; CC: Claudius’ cells; DC: Deiters’ cells; RM: Reissners’ membrane; SP: spiral prominence; SSZ: supra-strial zone; SV: stria vascularis. Scale bars: 50 μm.

**Figure 8:**
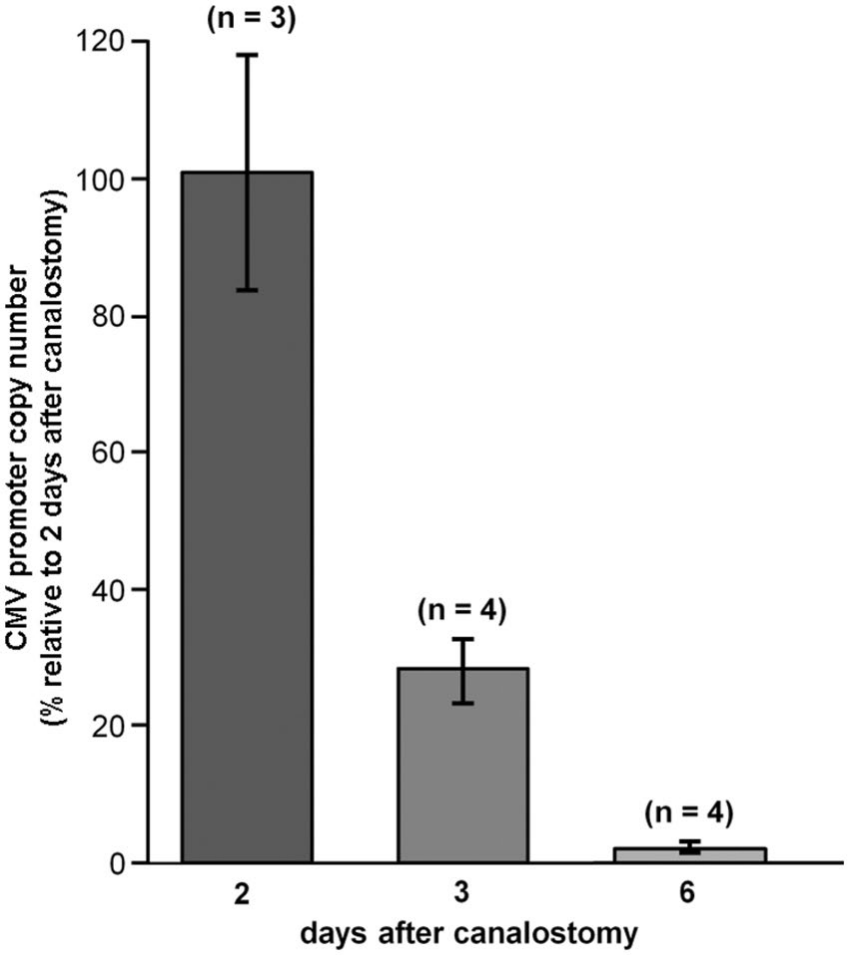
Quantification of BAAV genome following *in vivo* delivery at P4 via canalostomy to the mouse cochlea. CMV level was measured by q-PCR (see Methods) 2, 3 and 6 days after canalostomy and normalized to the level detected on day 2; error bars represent s.e.m.

The cochleae of these injected mice were studied by immunofluorescence four weeks after vector delivery via canalostomy. In order to avoid cross-reactivity of the anti Cx30 antibody with the residual Cx26 expressed by Cx30^Δ/Δ^ mice, we used an anti-GFP antibody that selectively targets the transgene product delivered by the BAAV vector. Confocal imaging showed extensive gap junction plaques formed by the Cx30GFP fusion protein at points of contacts between adjacent non-sensory cells of the sensory epithelium (Figs 6 and 7). Cx30GFP signals were also detected in the supra-strial zone (Fig. 7); we speculate that BAAV spread by transcytosis^57^ to accumulate in that zone traversing the lateral wall. Clearly, this viral fraction was lost as far as Cx30GFP expression in the critical sensory epithelium is concerned. Of note, all hair cells (both inner and outer) were preserved in this tissue after viral transduction and did not express Cx30GFP. These results indicate that recombinant Cx30GFP proteins traffic correctly to the plasma membrane of cochlear non-sensory cells not only *in vitro* ^4^ but also *in vivo* (Figs 6 and 7). However, Cx30GFP immunofluorescence signals in transduced cochlear epithelia were considerably weaker than the corresponding signals due to native Cx30 in age-matched non–injected wild type C57BL/6 N control mice (Figs 6 and 7). To address this issue, we quantified the viral genome copy number still present in the injected cochleae using primers specific for the CMV promoter. We found that the CMV signal was almost undetectable 6 days after transgene delivery via canalostomy (Fig. 8).

## Discussion

Viral transduction performed in adult *Gjb2* conditional knock out mice failed to correct hearing function^72, 73^, whereas an early intervention in newborn mice produced limited Cx26 reinstatement and only partial rescue of hearing^73^. Here, we have assayed the potential of BAAV vectors encoding Cx30 to rescue protein expression in Cx30 KO mice *in vivo* by transduction at P4. To our knowledge, this is the first report of a clear and widespread expression of exogenous Cx30 (tagged with GFP) that persisted in cells of the cochlear sensory epithelium four weeks after virus delivery via canalostomy. However, Cx30GFP expression was visibly lower than native Cx30 in wild type controls. This is partly due to the spread of BAAV particles to type V fibrocytes present in the supra-strial zone^74^, probably via transcytosis^57^ from scala media through the later wall, reducing the therapeutic index of the vector dose. Furthermore, we noted a decrease in BAAV genome level following transduction, which was almost undetectable already 6 days after delivery via canalostomy at P4. In contrast, BAAV-mediated gene delivery via canalostomy in wild type mice injected at P25 achieved extensive expression of a reporter gene (β−actin−GFP) that was maintained at high levels for at least one month.

Recent work with AAV serotypes indicates that restoration of hearing is within reach of cochlear gene therapy^53–55^. However, targeting the post-natal 3500 or so inner hair cells, or even the three to four-fold more numerous outer hair cells, is facilitated by the fact that both types of postnatal sensory cells are post-mitotically differentiated^75, 76^ and numerically stable from P0 onward^77–80^. Achieving early, stable and widespread expression of exogenous connexins in the far more abundant, heterogeneous and developmentally evolving populations of non-sensory cells in the sensory epithelium and lateral wall of the post-natal cochlea is more problematic. To better appreciate the inherent difficulties, one should consider that both sensory and non-sensory cells of the cochlear sensory epithelium originate from postmitotic progenitor cells, which undergo terminal mitosis between E12 and E14^75, 76^. A single row of inner hair cells develop from the lateral margin of the greater epithelial ridge (GER), whereas three to four rows of outer hair cells, and lateral non–sensory cells, develop from the lesser epithelial ridge (LER)^77–80^. At birth (P0), differentiated hair cells are found from the basal to the apical cochlear turn and thereafter form a life-long stable population^75–80^, however postnatal development continues^81^. Programmed cell death^82–85^ (type-I, or apoptosis, as well as type II, or autophagic cell death) plays a crucial role in tissue remodeling, specification of cell fate and differentiation^86–88^. Recently, autophagy-related genes *Becn1*, *Atg4g*, *Atg5* and *Atg9* were shown to be expressed in the mouse cochlea from late embryonic developmental stages (E18.5) to adulthood, and up-regulated as the postnatal inner ear gains functional maturity^89^. Whereas cytological changes that occur in the LER have not been studied extensively^75^, the abundant non-sensory cells that populate the GER are replaced by the far fewer cuboidal cells of the inner sulcus during the first two postnatal weeks^77–80^. Cells positive for activated caspase-3, one of the cysteine proteases that play essential roles in programmed cell death, were detected in the GER between P7 and P13, and appeared progressively along the cochlear duct from base to apex^90^. The GER persisted throughout all turns of the cochlea in 2-week-old mice lacking caspase-3, resulting in hyperplasia of supporting cells, degeneration of hair cells, and severe hearing loss, suggesting that caspase-3-dependent apoptosis is necessary for the development and formation of a properly functioning auditory system in mammals^91^. Administration of thyroid hormone (T3) to wild type mice on P0 and P1 advanced the overall program of apoptosis and remodeling by about 4 days, suggesting initiation of apoptosis by a receptor-mediated process in conjunction with other unknown signals^90, 92^. Given the extensive remodeling and cell turn over, near 100% transduction of non-sensory cells will be required in therapeutic intervention for connexin related disorders.

Various laboratories reported different values for the time required to reach maximal transgene expression level after viral delivery to the developing inner ear^72, 93^, possibly reflecting differences in the transduction pathway of the different AAV serotypes used in those studies. Recombinant AAV vectors are well known to remain mostly episomal and be lost quickly even after one round of cell replication^94^.

In summary, using three different viral vectors in wild type and transgenic mice, we show here that BAAV works efficiently as a gene manipulation tool and permits to control gene expression in non-sensory cells of the inner ear, *in vivo*. BAAV viral transduction provides efficient *in vivo* gene delivery to cochlear non−sensory cells via canalostomy, however rescue of DFNB1 phenotype requires early and stable expression of connexins, which is not afforded by the current generation of these viral vectors. Our results suggest that the persistence of BAAV-mediated gene replacement in the cochlea is limited by the extensive non-sensory cell loss, which occurs in the organ of Corti throughout postnatal development^88^. Future work is needed to improve vector production and reformulation that will allow higher concentrations of the BAAV vectors to achieve the near 100% transduction necessary in this application in the critical postnatal period that precedes acquisition of hearing in mice.

## Methods

### Transgenic mice and genotyping

All experimental protocols were approved by the Ethical Committee of Padua University (Comitato Etico di Ateneo per la Sperimentazione Animale, C.E.A.S.A.) Project n.58/2013, Protocol n. 104230, date 10/12/2013) and by the Italian Ministry of Health (DGSAF 0001276-P-19/01/2016). The methods were carried out in accordance with the relevant guidelines and regulations.

Mice of either sex were used. Cx26^*loxP/loxP*^ mice^67^ and Cx3^Δ/Δ^ mice^6^ were maintained on a pure C57BL/6 N background. Cx26^*loxP/loxP*^ mice were genotyped by screening for the presence of the *loxP* insertions on extracted mouse tail tips using the following primers:

Cx26F 5′-TTTCCAATGCTGGTGGAGTG-3′;

Cx26R 5′-ACAGAAATGTGTTGGTGATGG-3′.

A combination of three different primers was used to identify the Cx30 interruption due to LacZ insertion in Cx30^−/−^ mice

Cx30 F: 5′-GGTACCTTCTACTAATTAGCTTGG-3′,

Cx30 R: 5′-AGGTGGTACCCATTGTAGAGGAAG-3′,

Cx30lacZ: 5′-AGCGAGTAACAACCCGTCGGATTC-3′.

Genotyping of the *Cx30* ^*fl*^ allele was performed by PCR analysis using a primer binding in the Cx30 transcribed sequence and a primer binding upstream of the first loxP site

Gjb6R 5′-TTCCCTATGCTGGTAGAGTGCTTGT-3′;

Gjb6F 5′-GCAGTAACTTATTGAAACCCTTCACCT-3′.

Genotyping of the *Cx30* ^Δ^ allele was performed using the Gjb6F and a primer binding downstream of the third loxP site

Gjb6ΔR: 5′-CCCACCATCAAGGTTGAACT-3′.

### BAAV Production and Quantification

Hek293T cells, grown in DMEM/F12 supplemented with 5% FBS and 1% P/S, were transfected with three or four required plasmids (transgene vector, pAd12, and bovine adeno–associated virus (BAAV)–RepCap or AAV2–Rep plus BAAV-Cap). BAAVCx30GFP vector plasmids contained AAV-5 inverted terminal repeats (ITRs) whereas BAAVβ-actinGFP and BAAVCre-IRESGFP plasmids contained AAV–2 ITRs. Forty–eight hours after transduction, cells were harvested by scraping in TD buffer (140 mM NaCl, 5 mM KCl, 0.7 mM K_2_HPO_4_, 25 mM Tris-HCl pH 7.4) and the cell pellet was concentrated by low–speed centrifugation. Cells were lysed in TD buffer containing 0.5% deoxycholate and 100 U/ml DNase (Benzonase, Sigma) and incubated for 30 min at 37 °C. Following 10 min low speed centrifugation, viral particles were purified by CsCl gradient and dialyzed in DMEM/F12 (vehicle) with 10 K MWKO dialysis cassette (Pierce, Cat. No. 66383). Biological activity was confirmed on packaging cells^95^. Particle titers, determined by q-PCR, were in the range of 10^12^−10^13^ particles/ml. For viral titration, a dilution of the viral preparation was added to a q-PCR reaction mixture containing 1× SYBR Green Master Mix (Applied Biosystems/Applera, Milan, Italy) and 0.25 pmol/μl forward and reverse primers. Amplification was measured using a sequence detector (ABI 7700, Applied Biosystems). Specific primers for CMV were designed with the Primer Express program (Applied Biosystems):

CMV f 5′-CATCTACGTATTAGTCATCGCTATTACCAT-3′,

CMV r 5′–TGGAAATCCCCGTGAGTCA-3′.

Following denaturation at 96 °C for 10 min, cycling conditions were 96 °C for 15 s, 60 °C for 1 min for 40 cycles. The absolute quantification of the viral DNA in each sample was determined by generating a dilution series of a standard DNA.

### Canalostomy

For canalostomy, as well as all other surgical procedures described in this article, mouse body temperature was kept at 38 °C by a feedback-controlled heating pad. P25 mice were anaesthetized with an intraperitoneal injection of zolazepam (25 mg/g) and xylazine (10 mg/g) whereas P4 pups were anaesthetized with xylazine 0.45 µg/g and zolazepam 0.15 µg/g diluted in physiological solution. Supplemental doses were administered as needed. After induction of anesthesia, mice were placed under a dissection microscope and the posterior (P25) or lateral (P4) semicircular canal was exposed by a dorsal post-auricular approach. For P25 mice, a hole was made with the tip of a 33 G needle, softly removing a part of the bony shell of the canal. Bromophenol-blue (SIGMA, product number B−5525), Alexa594® conjugated wheat germ agglutinin (10 μg/ml; ThermoFisher, catalogue # W11262) dissolved in phosphate buffered saline (PBS) or viral solution diluted in DMEM/F12 (vehicle; Gibco^®^, catalogue # 11320074) were injected into the endolymphatic space (2.5 μl injected volume, 3 nl/s injection speed) with a micropump–controlled micro syringe (WPI, art.no. NANOFIL-100) equipped with a 36 G needle (WPI, art.no. NF36BV-2). For every transduction experiment, we injected ~10^9^ viral particles. To avoid fluid leakage during injection, the needle inserted in the semicircular canal of P25 mice was sealed with a drop of dental cement (Temrex Interface Light Cured Cavity Liner and Base, Product n. 7100), which was rapidly cured with blue light from a LED source (Mini Led, Satelec, F02641). Ten minutes after injection, the needle was slowly removed and the hole was closed with dental cement. To test the efficacy of delivery (Fig. 1), animals were sacrificed 10 minutes after injection and the excised cochlea was examined by light and confocal microscopy.

### *In vivo* electrophysiology

Endochlear potential was measured 30 days after surgery both in injected and contralateral ear. Mice were anaesthetized with 0.01 ml/g body weight of 20% urethane (SIGMA, product number 94300) and the potential difference between the scala media and a reference silver/silver chloride pellet under the dorsal skin was recorded^96^.

To record ABRs, mice were anaesthetized with an intraperitoneal injection of zolazepam (25 mg/g) and xylazine (10 mg/g) and submitted to clicks and tone pips at 8, 14, 20, 26, 32 kHz^62^.

### q-PCR

Total RNA was extracted from freshly dissected whole cochleae with RNeasy mini kit (Qiagen, Cat. No. 74106) and retrotranscripted with oligo–dT_12_–_18_ primers (ThermoFisher, catalogue # 18418012) using Omniscript RT kit (Qiagen, Cat. No. 205111). Total DNA was extracted from whole cochleae using GenElute Mammalian Genomic DNA miniprep kit (Sigma cat. # G1N10). q-PCR was performed on cDNA or DNA to amplify Cx26 and/or GFP and was normalized to GAPDH. Gene expression relative to GAPDH was estimated according to the method described by Pfaffl^97^. Amplification was carried out using Power SYBR Green (Applied Biosystems, Cat. No. 4367659) on the ABI 7700 sequence detection system equipped with ABI Prism 7700 SDS software (Applied Biosystems) through the following amplification cycles: 50 °C: 2′, 95 °C: 10′, 95 °C: 15′, 60 °C: 1′ (40 cycles). Primers used are as follows:

CMV f 5′-CATCTACGTATTAGTCATCGCTATTACCAT–3′,

CMV r 5′-TGGAAATCCCCGTGAGTCA-3′.

mCx26 f: 5′-TCACAGAGCTGTGCTATTTG-3′

mCx26r: 5′-ACTGGTCTTTTGGACTTTCC-3′

GFPf: 5′-TCCGCCATGCCCGAAGGCTA-3′

GFPf: 5′-CGGTTCACCAGGGTGTCGCC-3′

mGAPDHf: 5′-ATGTGTCCGTCGTGGATCTGAC-3′

mGAPDHr: 5′-AGACAACCTGGTCCTCAGTGTAG-3′

hGAPDHf: TGCACCACCAACTGCTTAGC

hGAPDHr: GGCATGGACTGTGGTCATGAG

### Immunohistochemistry and confocal imaging

Mice injected at P25 or P4 were sacrificed four weeks after injection. Cochleae were extracted and processed for immunohistochemistry as previously described^5^. Briefly, samples were fixed in 4% paraformaldehyde and decalcified in ethylenediaminetetraacetic acid (EDTA, 0.3 M). Specimens were included in 3% agarose dissolved in PBS and cut in 100 µm thickness steps using a vibratome (VT 1000 S, Leica). Tissue slices were permeabilized with 0.1% Triton X–100, dissolved in bovine serum albumin 2% solution. Cx30 and Cx26 were stained respectively with a rabbit polyclonal Cx30 and Cx26 selective antibodies (10 μg/ml, ThermoFisher, catalogue # 712200 for Cx30 and catalogue # 512800 for Cx26). A rabbit polyclonal GFP selective antibody (10 μg/ml, ThermoFisher, catalogue # A11122) was used to distinguish the exogenous fusion proteins from endogenous proteins. Secondary antibodies (10 μg/ml, Alexa Fluor® 488 goat anti-rabbit IgG, ThermoFisher, catalogue # A11008) were applied overnight at room temperature whilst F–Actin was stained by incubation with AlexaFluor 568 phalloidin (1U/ml, ThermoFisher, Cat. No. A12380) and nuclei were stained with 4′,6–diamidino–2–phenylindole (DAPI, ThermoFisher, Cat. No. D1306) (1:200). Samples were mounted onto glass slides with a mounting medium (FluorSave^TM^ Reagent, Merk, Cat. No. 345789) and analyzed using a confocal microscope (TCS SP5, Leica) equipped with an oil–immersion objective (40× HCX PL APO 1.25 N.A., Leica).

## Electronic supplementary material

Supplementary Information
