# Supplementary material for: In vivo genetic manipulation of inner ear connexin expression by bovine adeno-associated viral vectors
Source: Sci Rep. 2017 Aug 4;7:6567. doi: 10.1038/s41598-017-06759-y (PMC5544751; doi:10.1038/s41598-017-06759-y)
Supplement: Supplementary file 1 — Supplementary Information [file 41598_2017_6759_MOESM1_ESM.doc]

***In vivo* genetic manipulation of inner ear connexin expression by bovine adeno-associated viral vectors**

Giulia Crispino^1,2,#^, Fabian Galindo Ramirez^1,2,a,#^, Matteo Campioni^1,2,b^, Veronica Zorzi^3^, Mark Praetorius^4^, Giovanni Di Pasquale^5^, John A. Chiorini^5^,* and Fabio Mammano^1,2,3,6^,*

^1^*Venetian Institute of Molecular Medicine, Foundation for Advanced Biomedical Research, Padua, Italy*

^2^*Department of Physics and Astronomy "G. Galilei", University of Padua, Padua, Italy*

^3^*Department of Biomedical Sciences, Institute of Cell Biology and Neurobiology, Italian National Research Council, Monterotondo (RM), Italy.*

^4^*Department of Otolaryngology, University of Heidelberg Medical Center, Heidelberg, Germany;*

^5^*Molecular Physiology and Therapeutics Branch, National Institute of Dental and Craniofacial Research, National Institutes of Health, Bethesda, MD, USA;*

^6^*Shanghai Institute for Advanced Immunochemical Studies, ShanghaiTech University, Shanghai 201210, China;*

^a^Present address: Physiology Institute, Autonomous University of Puebla, Puebla, Mexico

^b^Present address: Department of Biochemistry and Biotechnology, University of Pavia, Pavia, Italy

^#^These Authors contributed equally to this article.

*Joint corresponding authors

Email

Giulia Crispino: [giulia.crispino@unipd.it](mailto:giulia.crispino@unipd.it)

Fabian Galindo Ramirez: [fabgalindoram@gmail.com](mailto:fabgalindoram@gmail.com)

Matteo Campioni: [matteo.campioni@unipv.it](mailto:matteo.campioni@unipv.it)

Mark Praetorius: [mark.praetorius@med.uni-heidelberg.de](mailto:mark.praetorius@med.uni-heidelberg.de)

Veronica Zorzi: [veronica.zorzi@cnr.ibcn.it](mailto:veronica.zorzi@cnr.ibcn.it)

Giovanni Di Pasquale: [gdipasquale@dir.nidcr.nih.gov](mailto:gdipasquale@dir.nidcr.nih.gov)

John A. Chiorini: [jchiorini@dir.nidcr.nih.gov](mailto:jchiorini@dir.nidcr.nih.gov)

Fabio Mammano: [fabio.mammano@cnr.it](mailto:fabio.mammano@cnr.it)


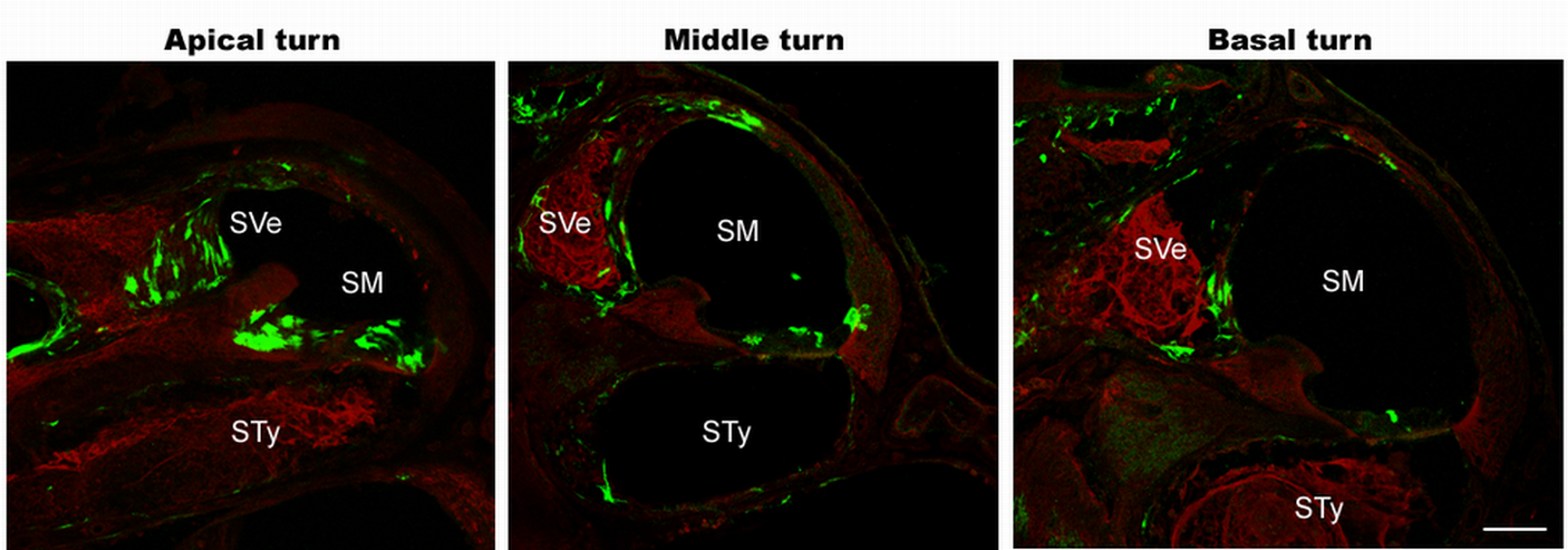


**Supplementary Figure 1.** **Confocal immunofluorescence imaging of cochlear cross–sections from mice injected at P25 with BAAVβ−actin−GFP by cochleostomy (apical, middle and basal turn of the cochlear duct).** Color code: β−actin−GFP, green; actin filaments, red**.** SVe: scala vestibuli; SM: scala media; STy: scala tympani. Scale bar: 100 µm.

**
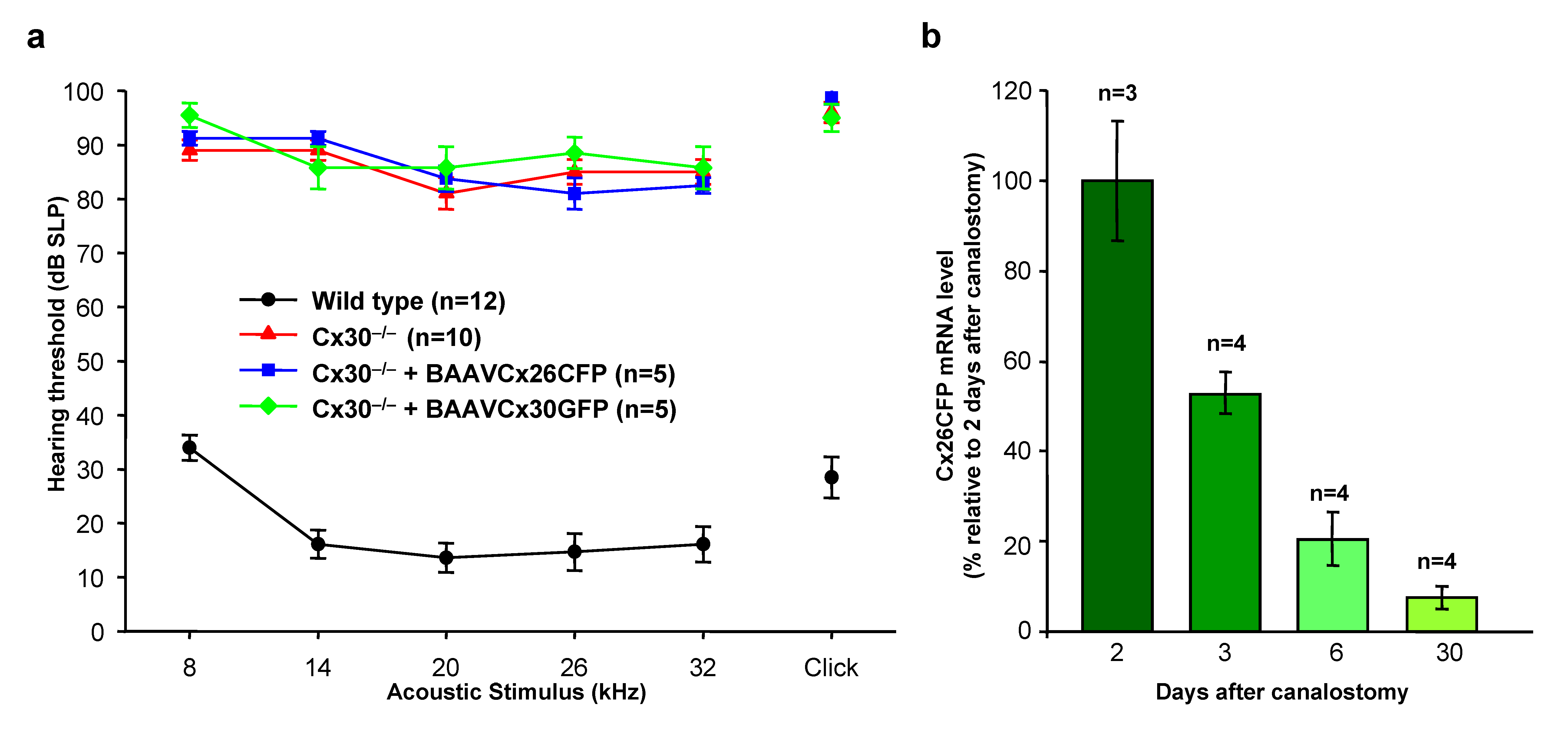
**

**Supplementary Figure 2.** ***In vivo* delivery of connexin genes to the inner ear of P4 Cx30^–/–^ mice.** (**a)** hearing thresholds from adult Cx30^–/–^ mice injected at P4 with BAAVCx26CFP or BAAVCx30GFP. **(b)** CFP mRNA level in cochleae from Cx30^–/–^ mice injected with BAAVCx26CFP quantified by q–PCR relative to the maximal expression detected 2 days after surgery.


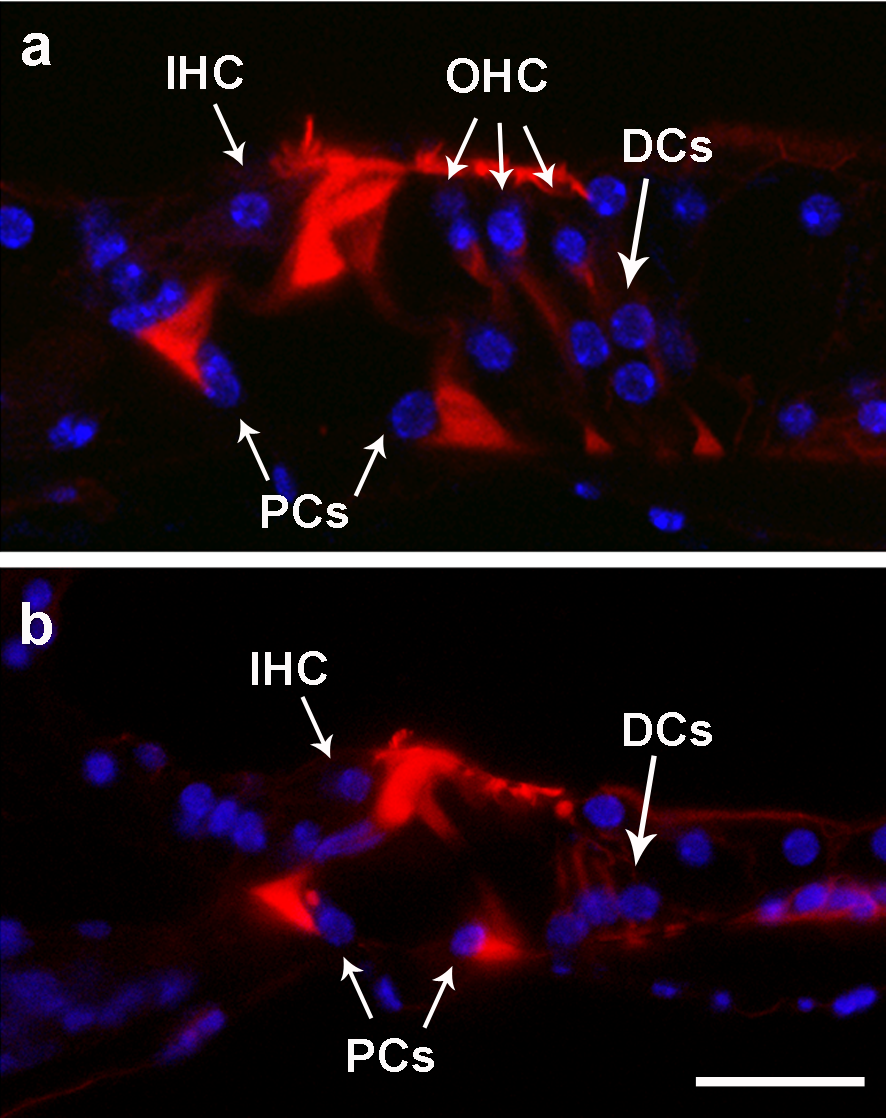


**Supplementary Figure 3.** Confocal immunofluorescence imaging of cochlear cross–sections from Cx30^Δ/Δ^ **(a)** and Cx30^−/−^ **(b)** mice. Color code: actin filaments, red; nuclei, blue. IHC: inner hair cell, PCs: pillar cells, OHC: outer hair cell, DCs: Deiters cells. Scale bar: 50 µm.

**List of abbreviations**

AAV: adeno−associated virus

ABR: auditory brainstem responses

BAAV: bovine adeno−associated virus

BC: Böttcher cells

BM: basilar membrane

Bo: bone

Ca^2+^: calcium

CC: Claudius’ cells

cDNA: complementary deoxyribonucleic acid

Cx26: connexin 26

Cx30: connexin 30

DAPI: 4’,6–diamidino–2–phenylindole

DC: Deiters’ cells

DMEM/F12: Dulbecco's Modified Eagle Medium/ Nutrient Mixture F−12

EDTA: ethylenediaminetetraacetic acid

EP: endocochlear potential

GAPDH: glyceraldehyde 3−phosphate dehydrogenase

GFP: green fluorescent protein

*GJB2*: gap junction beta−2 protein

*GJB6*: gap junction beta−6 protein

HC: Hensen’s cells

HCR: hair cell region

IHC: inner hair cell

IS: inner sulcus

ITRs: inverted terminal repeats

LM: spiral limbus

LW: lateral wall

MWKO: molecular weight cut−off

N.A.: numerical aperture

neo: neomycin

OHC: outer hair cell

OS: outer sulcus

OSL: osseous lamina spiralis

P4, P12, P25: postnatal day 4, 12, 25

PBS: phosphate buffered saline

PCR: polymerase chain reaction

PCs: pillar cells

q-PCR: quantitative polymerase chain reaction

RM: Reissner’s membrane

RNA: ribonucleic acid

SL: spiral ligament

SM : scala media

SP: spiral prominence

SSZ: supra-strial

STy: scala tympani

SV: stria vascularis

Sve: scala vestibuli

TM: tectorial membrane
